# Wear of enamel antagonist to gradient zirconia vs full strength monolithic zirconia: a randomized clinical trial

**DOI:** 10.1186/s12903-025-07566-y

**Published:** 2026-01-17

**Authors:** Sara Ismail Mohamed Hussein, Eman M Anwar, Omnia Nabil

**Affiliations:** 1 Department of Fixed Prosthodontics, Faculty of Dentistry, Cairo University, Cairo, Egypt; 2 Division of Fixed Prosthodontics, School of Dentistry, New Giza University, Giza, Egypt

**Keywords:** Zirconia, Monolithic zirconia, Wear, 3D superimposition

## Abstract

**Background:**

Monolithic zirconia restorations have emerged as a viable alternative to veneered zirconia restorations, eliminating the risk of veneer chipping due to their superior strength, fracture resistance, and aesthetics. New types of monolithic zirconia are becoming widely available, making it essential to clinically assess their wear behaviour and the effect on opposing enamel.

**Purpose:**

To evaluate the wear of enamel opposed by gradient monolithic zirconia compared to full-strength monolithic zirconia, and to assess the wear of both restorations.

**Methods:**

Twenty-six patients aged 21 to 35 years requiring a crown for endodontically treated molars were randomly divided into two groups. The comparator group (Zolid HT; Monolithic zirconia) included patients receiving full-strength monolithic zirconia crowns, while the intervention group (ZirCAD Prime; Multilayer zirconia) included patients receiving gradient monolithic zirconia crowns. After final cementation, a baseline direct scan was obtained for both arches, followed by additional scans at 6 months and 1 year post-treatment. These scans were imported into 3D software for superimposition to calculate the wear that occurred throughout the follow-up period for both restorations and the opposing enamel.

**Results:**

At the 6-month follow-up, the root mean square (RMS) values for Multilayer zirconia showed significantly higher wear of opposing enamel (75.6 μm) compared to Monolithic zirconia (61.18 μm), with a p-value of 0.0119. At the 1-year follow-up, Multilayer zirconia also exhibited significantly higher wear of opposing enamel (97.2 μm) compared to Monolithic zirconia (68.77 μm), with a p-value of 0.0046. Regarding restoration wear, RMS values at 6 months were comparable between Monolithic zirconia (82.65 μm) and Multilayer zirconia (72.16 μm), with a p-value of 0.6294. Similarly, at the 1-year follow-up, the RMS values remained statistically similar between Monolithic zirconia (89.47 μm) and Multilayer zirconia (73.57 μm), with a p-value of 0.4889.

**Conclusion:**

Gradient monolithic zirconia restorations were associated with greater enamel wear compared to Monolithic zirconia restorations, and the difference in wear depth became statistically significant over time. In terms of restoration wear, both types exhibited comparable material wear.

**Trial registration:**

This study was registered on clinicaltrials.gov with ID no. NCT04798300 on 28/1/2021.

## Introduction

Tooth wear is the non-carious, progressive loss of hard dental tissues resulting from complex and overlapping mechanical and chemical processes. While physiological wear is a normal consequence of aging and generally asymptomatic, excessive or accelerated wear can lead to structural degradation, sensitivity, and compromised function [1]. 

Clinically, tooth wear is categorized into attrition, abrasion, and erosion, terms first introduced by Hunter. Although these classifications are still widely used, they oversimplify the multifactorial nature of tooth surface loss. The oral cavity functions as a tribological system, where natural teeth and restorative materials interact under functional loading. Analysing tooth wear in this context requires a detailed understanding of material properties, particularly hardness, elastic modulus, and fracture resistance, to predict wear performance and compatibility between contacting surfaces [2–4]. 

Enamel, the hardest tissue in the human body with a Vickers Hardness Number of 250–360, undergoes physiological wear of about 30–40 μm annually in molars [5, 6]. To maintain occlusal balance and minimize antagonist wear, restorative materials should exhibit wear behaviour similar to enamel, making tribological compatibility crucial in material selection.

Monolithic zirconia restorations have emerged as reliable alternatives to veneered zirconia, especially in posterior regions due to their high fracture toughness and lower risk of chipping. However, clinical adjustments such as grinding and polishing can alter surface properties and affect wear behaviour [5]. Polished zirconia shows better compatibility with opposing enamel, whereas glazed and veneered ceramics tend to cause more enamel wear due to increased surface roughness. Therefore, proper surface finishing after adjustments is essential to minimize abrasiveness, as it is more directly related to enamel wear than the material’s hardness [7–11].

Zirconia-based ceramics have evolved through successive generations, resulting in variations in microstructure, yttria content, and phase distribution that influence their mechanical and tribological properties. In contemporary dental materials science, zirconia ceramics are broadly categorized according to their yttria content into three main types: 3 mol% yttria-stabilized tetragonal zirconia polycrystal (3Y-TZP), 4 mol% yttria-partially stabilized zirconia (4Y-PSZ), and 5 mol% yttria-partially stabilized zirconia (5Y-PSZ) [12–14]. 

Differences in phase composition and translucency between zirconia materials suggest variations in their wear behaviour. While the wear performance of 3Y-TZP is well established, limited clinical data exist for high-translucency zirconias with higher cubic phase content, such as 4Y-PSZ and 5Y-PSZ. Few studies have directly compared these materials [15–20]. Wear evaluation methods range from subjective indices to objective techniques like profilometry, micro-CT, and digital superimposition [21–24]. Advances in intraoral scanning and CAD-CAM workflows now enable accurate, non-invasive quantification of surface wear using STL superimposition and colour-mapped deviation analysis [25, 26]. 

Despite the increasing use of monolithic zirconia, limited clinical evidence compares the wear behaviour of gradient multilayer zirconia and full-strength 3Y-TZP. Most existing studies focus on surface finishing, with few addressing compositional differences in vivo. Given the rising adoption of multilayer systems for aesthetic and functional restorations, this study aimed to provide 12-month clinical data on enamel and restoration wear to guide material selection in daily practice.

The study’s null hypothesis was that there would be no difference in the wear of enamel opposing both tested restorations and that there would be no difference in wear between Monolithic Zirconia and Multilayer Zirconia.

## Materials and methods

### Study design

The current study was a randomized controlled clinical trial (RCT) involving twenty-six participants allocated into two groups based on the material used. The Intervention Group received full coverage gradient zirconia crowns, while the comparator group received full strength monolithic zirconia crowns (Table 1). Participants of both groups received twenty-six single posterior full coverage crowns (1st molar). Participants were recruited from the Prosthodontics Department at Cairo University’s Faculty of Dentistry. Before starting clinical procedures, the treatment plan was thoroughly explained to each participant, and informed consent was obtained. All clinical interventions were performed by a single experienced clinician to ensure consistency.

**Table 1:** Material used

Material name	Composition (in % by weight)	Manufacturer
IPS e-max ZirCAD Prime zirconia	88.0-95.5% (ZrO₂)	Ivoclar Vivadent (Germany)
	>4.5. %-≤7.0% (Y₂O₃)	
	≤1.0% (Al₂O₃)	
Monolithic Zolid HT Pre-shaded	≥99.0 (ZrO₂)	AMMANGIRBACH (Germany)
	6.0–7.0 (Y₂O₃)	
	≤O.5 (Al₂O₃)	

Abbreviations: *Al₂O₃* Aluminum oxide, *ZrO₂* Zirconium dioxide, *Y₂O₃* Yttrium oxide

**Fig. 1:**
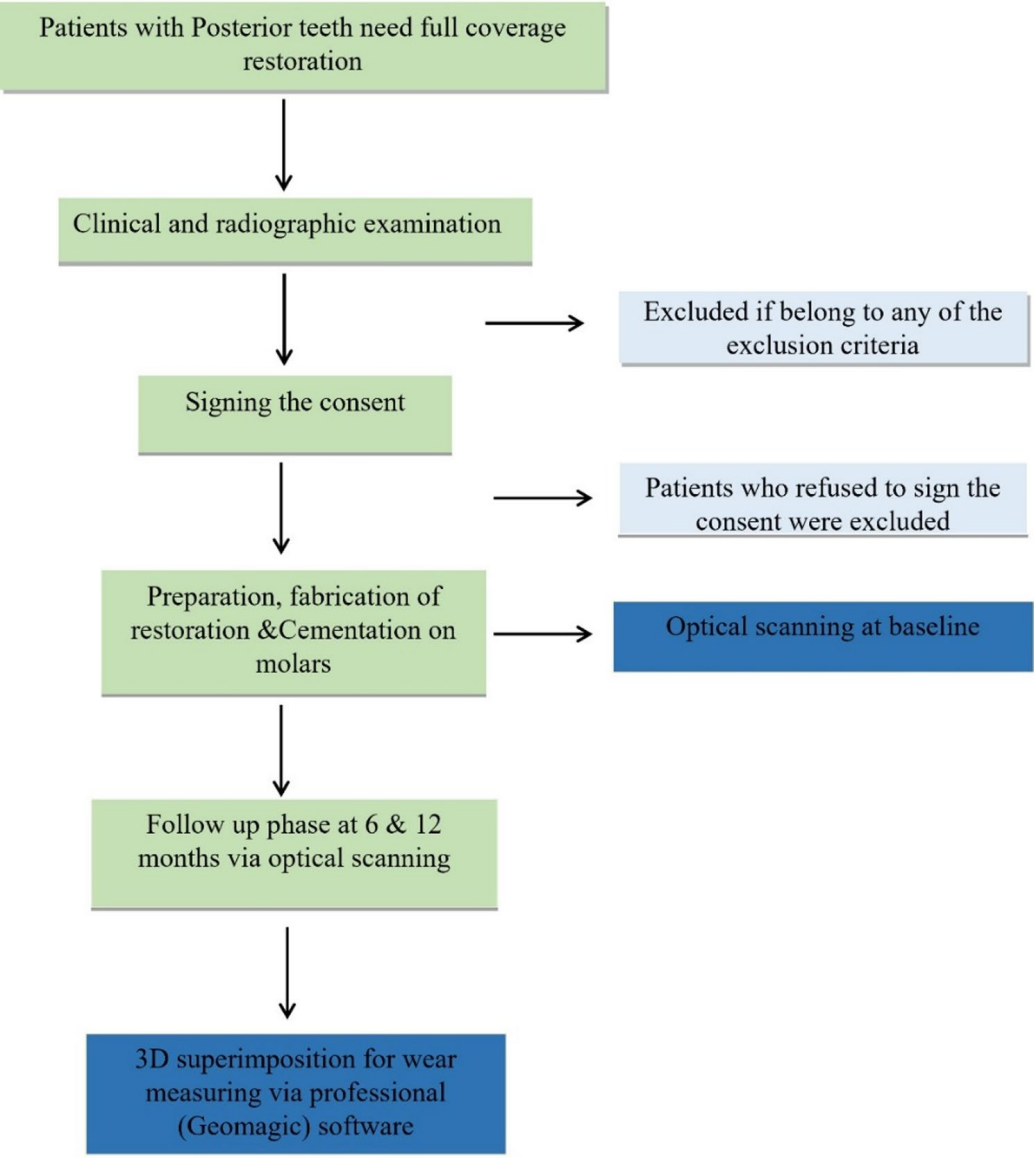
Treatment plan

Participants were randomly assigned to the two groups in a 1:1 allocation ratio based on the material used, utilizing a computerized randomization tool (www.randomizer.org) [27]. A fixed block size of 2 was employed to ensure balanced group sizes. Sequentially numbered opaque sealed envelopes containing pre-generated group assignments were used for allocation concealment. Randomization was conducted by one of the main supervisors (O.N.), who released the envelopes to the operator at the time of tooth preparation. The study spanned one year, encompassing the recruitment phase through to the final follow-up evaluations (Fig. 1).

The primary outcome for this study was enamel wear (µm) after 12 months. Sample size estimation was based on data reported by Mundhe, which indicated a mean wear of molars opposing zirconia crowns at 12 months of 127 ± 5.03 μm [28]. An expected mean difference of 6.5 μm between the intervention and control groups was determined based on expert clinical judgment. Assuming a significance level (α) of 0.05 and a power (1 − β) of 80%, the minimum required sample size was calculated to be 10 subjects per group (total *N* = 20). To account for an anticipated dropout rate of 25%, the sample size was increased to 13 participants per group. Calculations were performed using Power and Sample Size Calculations software, version 3.0.

### Inclusion criteria

All patients were required to:

1. Be between 21 and 35 years old and able to read and sign the informed consent document.
2. Be free of active periodontal or pulpal diseases.
3. Be psychologically and physically capable of undergoing conventional dental procedures.
4. Require a single coverage restoration in the posterior area.
5. Have intact opposing antagonists.
6. Be able to return for follow-up examinations and evaluations.

### Exclusion criteria

1. Patients younger than 21 or older than 35 years.
2. Patients with severe clenching or bruxism habits.
3. Patients with active resistant periodontal disease.
4. Patients with poor oral hygiene or who are uncooperative.
5. Pregnant women.
6. Patients in the growth stage with partially erupted teeth.
7. Patients with psychiatric problems or unrealistic expectations.
8. Lack of opposing dentition in the area of interest.
9. Presence of dental prostheses in the opposing arch in the area of interest.

A total of twenty-six patients (10 males and 16 females) aged between 21 and 35 years were selected for this study. This age range was chosen to minimize the potential influence of age-related physiological changes on occlusal forces, as bite force has been reported to decline with age, particularly after 45 years in males and after 25 years in females [29]. However, gender differences in bite force were not specifically considered, as the primary aim of the study was to evaluate tooth wear, not to analyse bite force variation between sexes. All crowns were placed on first molars, ensuring uniformity in tooth type and occlusal function across the study groups.

### Clinical procedures

#### Preoperative examination

A comprehensive evaluation was conducted to confirm normal occlusion. The extraoral examination assessed facial symmetry, vertical dimensions, and evaluation of the temporomandibular joint (TMJ) and muscles of mastication, while the intraoral assessment focused on remaining tooth structure, gingival and periodontal health, and any signs of wear.

To control for confounding factors influencing enamel wear, participants were screened for signs and symptoms of bruxism. Probable bruxism, defined as a combination of self-reported symptoms and clinical findings, was used as the threshold for exclusion [30, 31]. 

All participants completed a standardized, validated self-report questionnaire assessing the presence of awake or sleep bruxism. Questions included reports of teeth grinding or clenching during sleep, awareness of jaw tension or clenching during the day, history of morning headaches, and reports from bed partners regarding nocturnal grinding. A clinical examination was also performed.

The clinical assessment included evaluation of:

1. Non-physiological wear facets inconsistent with the participant’s age or dietary habits.
2. Hypertrophy of the masseter muscles.
3. Tongue or cheek indentations.
4. Tenderness in the masticatory muscles or temporomandibular joints.
5. Presence of occlusal splints or a history of bruxism treatment.

Participants demonstrating both self-reported symptoms and one or more clinical signs were diagnosed with probable bruxism and excluded from the study. Additionally, individuals with occlusal appliances or those exhibiting advanced localized enamel wear inconsistent with physiological patterns were also excluded. This ensured that enamel wear recorded during the study was not influenced by parafunctional habits.

#### Tooth preparation and crown fabrication

All clinical and laboratory procedures adhered strictly to a standardized clinical and technical protocol. Before crown preparation, lost tooth structure was replaced with a glass fiber post and a fiber-reinforced core build-up using adhesively retained composite resin (Build-it™ ER, Pentron Clinical Technologies, USA). The teeth were prepared with 1 mm circular reduction and 1.0–1.5 mm occlusal reduction, featuring a pronounced circumferential chamfer finish line.

Final impressions were taken using Vinyl Polysiloxane addition silicone (Panasil, Kettenbach Dental, Germany) in a perforated metal tray. A two-step impression technique was employed to enhance accuracy [32]. Master casts were poured using type IV dental stone, and the casts were scanned with an extraoral scanner (DOF Swing HD, Nevada). The 3D virtual models were imported into EXOCAD software, where the imported Standard Tessellation Language (STL) data was used to design the final restoration. Dry milling of both monolithic zirconia (Zolid HT, AMMANGIRBACH) and multilayer zirconia (ZirCAD Prime, Ivoclar Vivadent) restorations was then performed using a five-axis milling machine (DWX-52D, USA).

A try-in was conducted with the final restoration to assess marginal fit, contacts, contour, and occlusion. If any high spots were present, occlusal adjustments were made to achieve the final occlusal scheme. Proper occlusal contact was confirmed during the try-in stage, and no further adjustments were made following the final polishing protocol and adhesive cementation.

#### Restoration finishing technique

The final polishing step for the occlusal surface was performed using a polishing kit (DIACERA HP DIASYNT POLISHING, EVE Technik GmbH, Germany) to achieve a high polish. The polishing protocol involved using two rubbers for 60 s each: first, a coarse light green rubber was used for smoothing and pre-polishing, followed by the fine pink rubber for a high-gloss finish. Next, a goat hair brush [33] and a diamond polishing paste (PEARL SURFACE Z, Kuraray Noritake, Japan) were applied for an additional 60 s. Prior to this polishing step, staining and glazing of buccal surfaces were performed to enhance the natural appearance of the restorations. A single-step, uniform layer of glaze paste and stains (Noritake CZR Stain & Glaze, Noritake Co., Ltd., Nagoya, Japan) was applied approximately 2 mm away from the cusps to avoid the occlusal surface and articulation areas. No additional firing was conducted after this application. The fitting surface of the zirconia crown was sandblasted with aluminum oxide (Al₂O₃, particle size 50 μm, at a pressure of 0.1 MPa, with a 15 mm step-over distance for 10 s), followed by a second try-in to ensure perfect occlusion.

#### Surface treatment and cementation

**Fig. 2:**
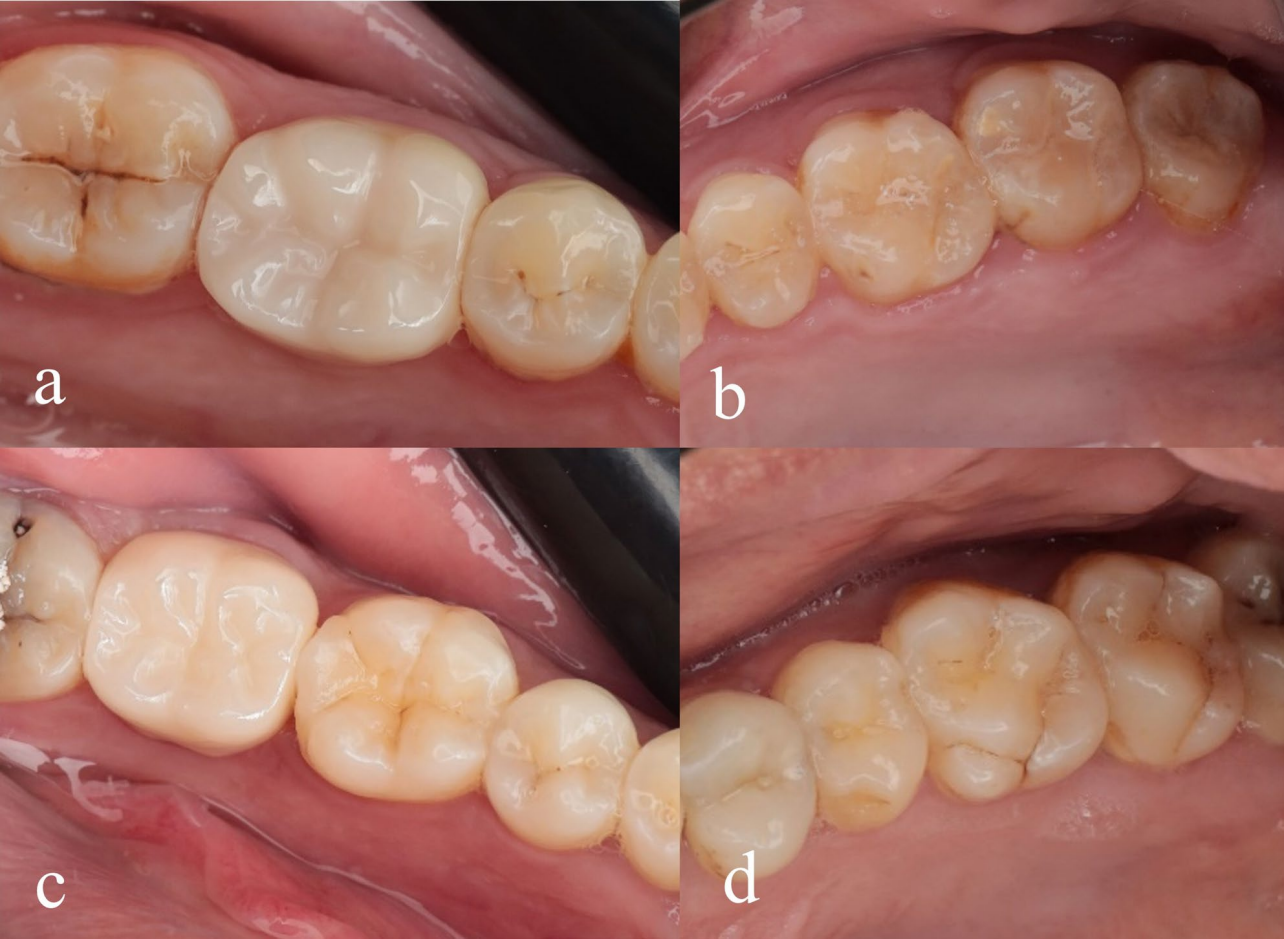
(**a**) Monolithic zirconia crown; (**b**) Antagonist enamel; (**c**) Multilayer zirconia crown; (**d**) Antagonist enamel

The fitting surface of the crown was cleaned using a cleaning paste (Ivo Clean, Ivoclar Vivadent, Germany), which effectively removes saliva contaminants. Subsequently, zirconia primer (Z-Prime Plus, BISCO, USA) was applied to achieve optimal bonding. Final cementation was performed using the luting adhesive resin cement technique (Breeze resin cement, Pentron, USA) [34, 35] (Fig. 2).

### Data collection

Two blinded evaluators assessed the outcomes. The baseline scan was performed on the day of cementation. At the 6-month and 1-year follow-up periods, patients returned for intraoral scans. During these sessions, the teeth were polished, and an intraoral scanner (TRIOS 4, Denmark) collected scans of both arches. The STL files representing the dental arch with restorations and the antagonist from both baseline and follow-up visits were imported into Geomagic Control X 2022 1.0, a 3D inspection and metrology software for measuring wear.

A superimposition process aligned the STL files from the two follow-up scans with the baseline scan using initial alignment and best-fit alignment tools. To ensure precise superimposition and accurate wear measurements, a 3D segmentation step excluded irrelevant data, particularly soft tissue and teeth outside the area of interest. For occlusal wear analysis, the crowns of the teeth were segmented into occlusal surfaces and axial walls, with wear measurements focused exclusively on the occlusal surfaces [8]. 

Quantitative wear was computed in root mean square (RMS) using the 3D compare tool with tolerance 0.05. Tooth wear was quantified by calculating the RMS of point-to-point deviations within the defined occlusal surface area. The RMS value represents the average 3D surface deviation and was expressed in micrometers (µm). Geomagic calculates the RMS using the following formula: RMS=$\:\sqrt{\frac{1}{n}\sum\:_{i=1}^{n}\left(d_{i}\right)^2}$ ; n = number of measured points evenly distributed over the selected surface area and d_i_ = distance between a point on the follow-up scan and the corresponding point on the baseline scan [36]. 

Deviations are measured by sampling numerous surface points on the follow-up scan. For each point, the software calculates the shortest perpendicular distance to the baseline scan. These distances are classified as follows: Positive if the point on the follow-up scan is above the baseline, indicating saliva or inaccurate scanning; Negative if the point is below the baseline, indicating wear; and Zero if both surfaces coincide at that point [8]. 

**Fig. 3:**
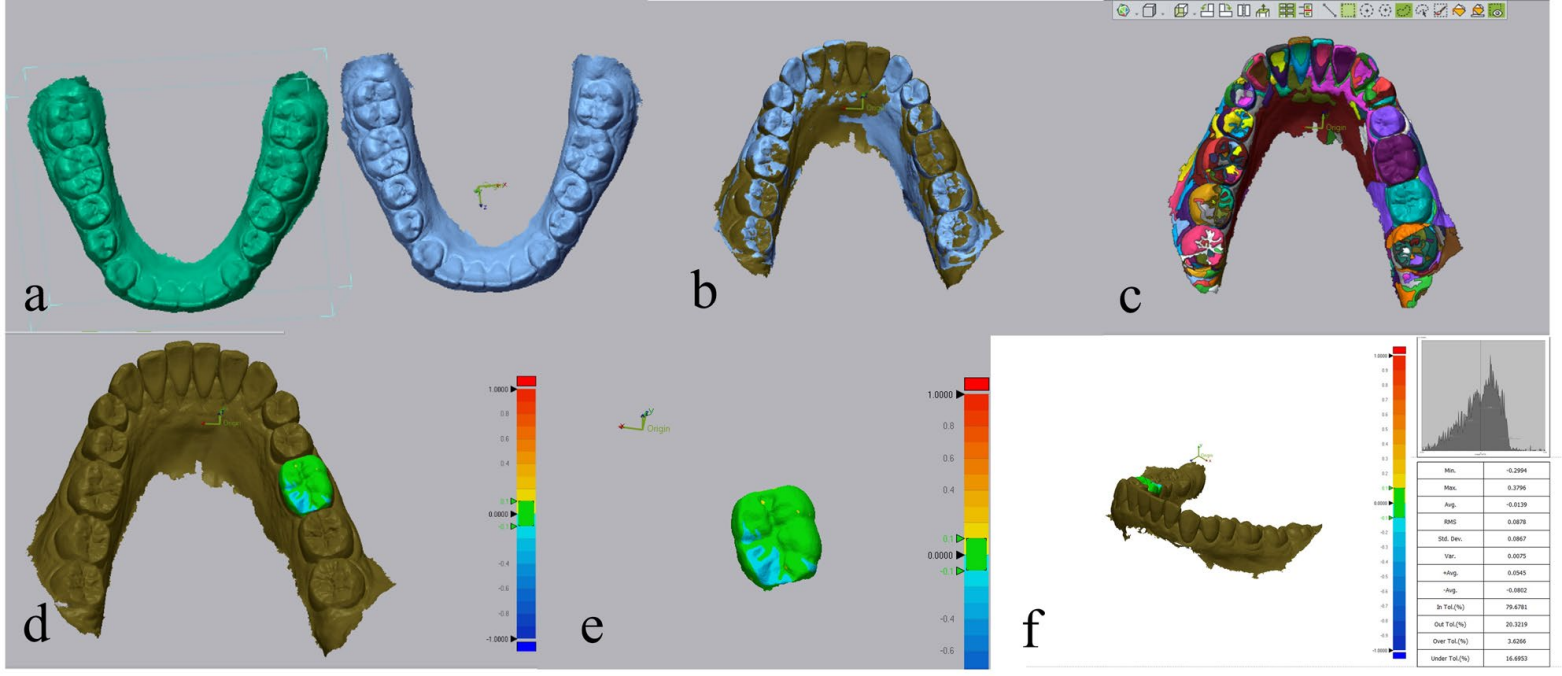
Geomagic superimposition of restoration: (**a**) Dental arch scans at baseline and 6 months follow-up; (**b**) Initial alignment step; (**c**) Segmentation step; (**d**) 3D comparison between scans using color map where blue color represents areas of restoration wear; (**e**) Isolation of the crown; (**f**) Auto-generated software report representing wear of the restoration in RMS and average negative

**Fig. 4:**
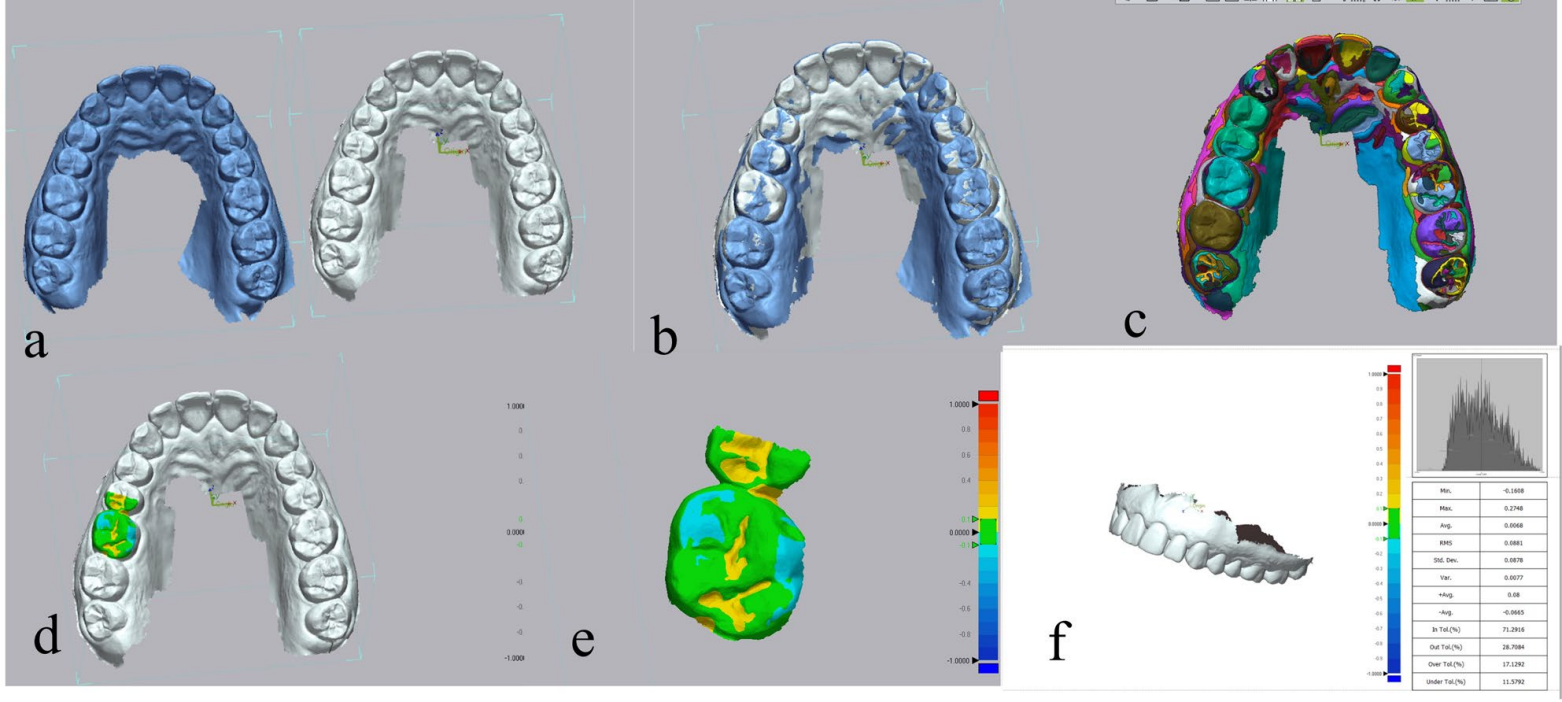
Geomagic superimposition of antagonist enamel: (**a**) Dental arch scans at baseline and 6 months follow-up; (**b**) Initial alignment step; (**c**) Segmentation step; (**d**) 3D comparison between scans using color map where blue color represents areas of antagonist enamel wear; (**e**) Isolation of the antagonist; (**f**) Auto-generated software report representing wear of the antagonist enamel in RMS and average negative

Qualitative wear assessment used a colour map: green indicates ideal alignment without surface wear, blue indicates areas of wear, and yellow indicates potential inaccuracies due to intraoral scanning, saliva, or food debris on crown surfaces (Figs. 3 and 4).

### Statistical analysis

Paired t-tests were employed to assess differences in measurements obtained at two time points (6 months and 1 year) within the same group, specifically to evaluate changes in restoration wear over time. While Independent t-tests were used to assess differences in mean outcomes between the two groups at 6 months and 1 year. Statistical significance was set at *p* < 0.05. All statistical analyses were conducted using IBM SPSS Statistics, version 23.0 (Armonk, NY: IBM Corp.).

## Results

**Fig. 5:**
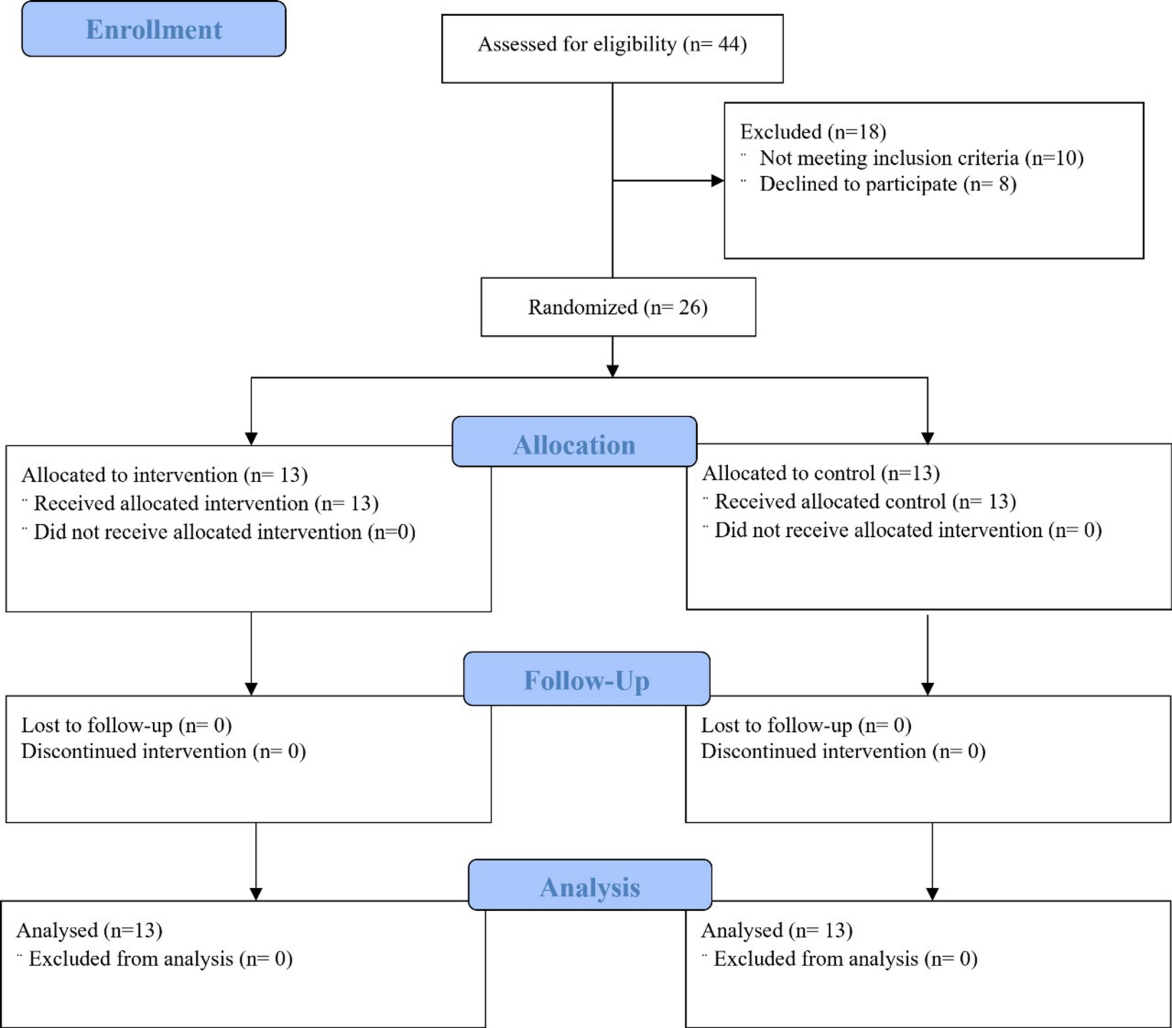
CONSORT flow chart

The distribution of patients across the study stages was documented according to the CONSORT flow diagram, with “n” indicating the number of patients at each stage (Fig. 5).

Both RMS and average negative values were calculated.

### Antagonist enamel wear

The findings revealed significant differences between the two groups. Both RMS and average negative values were significantly higher for Multilayer zirconia restorations compared to Monolithic zirconia. At the 6-month follow-up, the RMS for Multilayer zirconia was 75.6 μm, while it was 61.18 μm for Monolithic zirconia (*p* = 0.1081). At the 1-year follow-up, the RMS value for Multilayer zirconia was significantly higher at 97.2 μm compared to 68.77 μm for Monolithic zirconia (*p* = 0.0046).

Analysis of the average negative values, representing wear depth, also revealed statistically significant differences between the two groups. At both the 6-month (*p* = 0.0446) and 1-year (*p* = 0.0021) follow-ups, the average negative value was significantly lower (more negative) for Multilayer zirconia restorations compared to Monolithic zirconia. Both RMS and average negative values indicate that enamel wear increased with depth when opposing Multilayer zirconia restorations compared to Monolithic zirconia restorations (Table 2).

**Table 2:** Independent-t test analysis of enamel wear opposing monolithic zirconia and multilayer zirconia

		Monolithic zirconia		Multilayer zirconia		P-value
		M	SD	M	SD	
Root mean square (µm)	6 months follow up	61.18	15.83	75.6	21.83	0.1081 NS
	1 year follow up	68.77	16.9	97.2	22.1	0.0046*
Average Negative	6 months follow up	-39.74	15.23	-59.57	24.74	0.0446*
	1 year follow up	-40.91	13.6	-79.37	30.94	0.0021*

Abbreviations: *SD* Standard deviation, *M* Mean, *P* Probability level

### Restoration wear

The data presents a comparative analysis of wear between Monolithic zirconia and Multilayer zirconia groups during the 6-month and 1-year follow-up periods.

The independent t-test analysis revealed no statistically significant differences in wear characteristics between the two materials. At 6 months, the RMS values were similar, with Monolithic zirconia at 82.65 μm and Multilayer zirconia at 72.16 μm (*p* = 0.6294). At the 1-year follow-up, the RMS values remained comparable, with Monolithic zirconia at 89.47 μm and Multilayer zirconia at 73.57 μm (*p* = 0.4889). The average negative values, representing wear depth, also showed no statistically significant differences between the two materials at both the 6-month (*p* = 0.6270) and 1-year (*p* = 0.5301) marks (Table 3).

**Table 3:** Independent-t test analysis of wear of monolithic zirconia and multilayer zirconia restorations:

		Monolithic zirconia		Multilayer zirconia		P-value
		M	SD	M	SD	
Root mean square (um)	6 months follow up	82.65	59.81	72.16	31.44	0.6294 NS
	1 year follow up	89.47	59.54	73.57	38.99	0.4889 NS
Average Negative	6 months follow up	-74.93	58.49	-64.7	29.35	0.6270 NS
	1 year follow up	-80.19	56.91	-66.49	36.62	0.5301 NS

## Discussion

Tooth wear is a complex and irreversible process marked by the gradual loss of tooth structure, influenced by various factors. This phenomenon typically arises from direct contact between teeth, dental restorations, or prostheses during chewing or due to abnormal oral habits [6]. 

When selecting a restorative material, it is essential to consider the wear that occurs between enamel and restorations. As monolithic zirconia restorations become increasingly common and new types are introduced, their wear behaviour and effect on opposing enamel must be assessed clinically [12]. 

While laboratory simulations provide valuable insights, they do not fully replicate the complex wear dynamics observed in the oral environment. Therefore, the assessment of dental materials, particularly zirconia ceramics, cannot rely solely on in vitro studies.

Despite its importance, quantitative data on the absolute amount of in vivo tooth wear is limited. Contradictory findings regarding tooth wear progression are common, largely due to significant methodological variations in studies [25]. This study employed a direct measurement approach for assessing clinical wear, contrasting with previous investigations that relied on replicas. It utilized an intraoral scanner to capture the antagonist enamel and fabricated restorations, minimizing potential inaccuracies associated with the replication process.

Although extensive research has evaluated the wear behaviour of zirconia-based restorations, most studies focus either on surface treatments or comparisons between zirconia and non-zirconia ceramics. Few clinical investigations have directly compared full-strength monolithic zirconia with strength-graded zirconia under in vivo conditions. Moreover, even fewer studies incorporate standardized surface finishing protocols and quantitative 3D wear analysis over time. This study addresses that gap by providing one-year follow-up data on both antagonist enamel and restoration wear, offering clinically relevant insights into how zirconia formulation influences wear behaviour, particularly in younger adult populations.

The null hypothesis of no difference in wear characteristics between full-strength and strength-gradient zirconia was partially rejected. While no significant differences were found in restoration wear, there were statistically significant differences in antagonist enamel wear, suggesting that internal material composition and structure may influence how zirconia interacts with opposing dentition.

The influence of 3Y-TZP monolithic zirconia on antagonist enamel wear has been assessed in research. Lohbauer and Reich evaluated the wear of antagonist occlusal surfaces opposing clinically placed monolithic zirconia premolar and molar crowns and reported that 3Y-TZP restorations exhibited acceptable enamel wear after 24 months in vivo [20]. A recent 5-year prospective study further supports these findings, showing that polished monolithic 3Y-TZP resin-bonded fixed partial dentures caused only slightly higher enamel wear than natural enamel contacts, with no detectable wear on the zirconia itself [24]. Overall antagonist wear remained clinically acceptable, reinforcing that properly polished monolithic 3Y-TZP zirconia is enamel-friendly over long-term function.

Despite extensive research, there remains a lack of in vivo trials that directly compare monolithic zirconia with strength-graded zirconia regarding antagonist enamel wear. However, the findings of antagonist enamel wear of monolithic zirconia in the present trial align with the results of De Angelis et al. (2022) who reported significantly greater antagonist wear with 5Y-PSZ compared to 3Y- and 4Y-based zirconia. In their study, zirconia specimens were opposed by type III gold alloy cusps, which are documented to exhibit enamel-like wear behavior. Under these conditions, 5Y-PSZ produced markedly higher wear on the enamel-analog material, reinforcing the evidence that increased cubic-phase content and the associated loss of transformation toughening contribute to greater antagonist wear. These findings suggest that strength-gradient materials typically 5Y-PSZ-based may compromise mechanical resilience in exchange for enhanced translucency [16]. 

The superior microstructure and mechanical integrity of full-strength zirconia likely minimize surface degradation and microfracture formation, contributing to its protective effect on opposing enamel. In contrast, strength-gradient zirconia, engineered for enhanced aesthetics and stress distribution, may have microstructural inconsistencies or variable surface hardness that promote micro-abrasion or roughening factors, exacerbating enamel wear [15]. 

On the other hand, Zhang et al. (2019) reported no significant differences in enamel wear among 3Y, 4Y, and 5Y zirconias, emphasizing surface roughness over zirconia composition as the primary determinant of antagonist wear. They employed a direct comparative method, using a steatite ball as the antagonist, a common model in dental wear studies; however, it does not fully replicate the mechanical properties of natural enamel, including elastic modulus and fracture behavior. In addition to the high number of cycles and elevated temperature in water that simulated an accelerated, worst-case wear scenario, enhancing the relevance of their findings [17]. 

Similarly, Rosentritt et al. (2020) observed no significant differences in enamel wear among zirconia generations in vitro. However, like Zhang’s study, steatite spheres were used as antagonists rather than natural enamel, and steatite differs from enamel in hardness, fracture toughness, and wear patterns, which can alter the relative aggressiveness of zirconia surfaces. Additionally, the study utilized a pin-on-block two-body wear setup, which does not fully reproduce the multidirectional sliding, lubrication, and intermittent loading characteristic of enamel contact during mastication [18]. 

Contradicting both Zhang and Rosentritt, Madanshetty et al. (2023) reported greater antagonistic enamel wear with 3Y-TZP compared to 5Y-PSZ. In their study, three generations of zirconia ceramics were evaluated using standardized zirconia discs placed in contact with extracted human premolars and tested in a two-body wear machine. The specimens were subjected to a constant load of 5 kg and underwent 10,000 wear cycles, with continuous application of artificial saliva to simulate intraoral conditions. The authors attributed the increased enamel wear associated with 3Y-TZP to its higher tetragonal phase content and the presence of Al₂O₃ particles, which contribute to greater material hardness and potentially increased abrasiveness [19]. However, current evidence indicates that antagonist enamel wear is primarily determined by the surface characteristics of zirconia restorations and is independent of the material’s intrinsic hardness, demonstrating that variations in hardness among different zirconia generations do not reliably predict enamel wear [9–11]. This discrepancy may be explained by the unspecified finishing protocol in Madanshetty et al.’s study, particularly whether the zirconia surfaces were glazed or polished.

This spectrum of results reinforces the importance of clinical studies, as laboratory simulations may overestimate or underestimate wear behaviour by omitting dynamic oral variables. In vivo data provide valid evidence to guide material selection in clinical practice.

The findings regarding restoration wear in the present study align with those of Kwon et al. (2018), who demonstrated that both 3Y-TZP and 5Y-PSZ exhibit excellent wear resistance. Their study emphasized that ceramic wear often originates from microfractures or surface chipping during occlusal contact, creating surface roughness that leads to increased enamel abrasion. Notably, they observed that despite the lower flexural strength of 5Y-PSZ compared to 3Y-TZP, no surface fracturing or roughening occurred during wear simulation, suggesting that 5Y-PSZ maintains a stable and smooth surface under functional loading [20]. These results support the clinical performance of newer high-translucency zirconias when appropriately polished and finished.

In contrast, our findings differ from those of De Angelis et al. (2022), who reported compromised wear resistance in 5Y-PSZ compared to 3Y-TZP and 4Y-PSZ. Their explanation focused on the structural changes induced by increased yttria content, which stabilizes the cubic phase at the expense of the tetragonal phase. While this enhances translucency by reducing birefringence and light scattering, it also compromises mechanical properties, particularly flexural strength and fracture toughness, due to the elimination of the transformation toughening mechanism inherent to 3Y-TZP. According to their in vitro study, these changes may lead to a higher susceptibility to surface damage and subsequent wear [16].

The discrepancy between the present study’s findings and those of De Angelis et al. may stem from the the restoration-to-restoration test setup likely influenced wear patterns, as contact between two restorative materials differs from clinical restoration-to-enamel interactions, which may produce higher antagonist wear.Therefore, clinical trials, such as the present study, provide more valid data regarding material performance under realistic masticatory conditions..

### Limitations of the study

This study has several limitations. First, the relatively short clinical observation period may limit the ability to capture long-term wear behaviour; extended follow-up would enhance the reliability of the findings. Additionally, the study was limited to single crowns, and results may differ with multiple units or long-span fixed partial dentures (FPDs).

Although intraoral scanning with the Trios 4 scanner and Geomagic software offers a high-resolution, non-invasive method for wear detection, minimal changes over a short period may approach the system’s detection threshold. Factors such as saliva, patient movement, and scan quality could also introduce variability.

Furthermore, the use of whole-occlusal best-fit alignment without excluding worn areas may slightly limit the precision of localized wear detection, although this method is widely accepted in clinical research. Future studies could incorporate occlusal contact mapping or region-specific alignment to improve accuracy.

Lastly, evaluating the impact of different surface finishing protocols and opposing restorative materials would provide a more comprehensive understanding of wear behaviour under varied clinical conditions.

## Conclusions

Within the limitations of this clinical study, both monolithic zirconia and strength-gradient zirconia restorations demonstrated comparable and clinically acceptable levels of material wear over 12 months. However, significant differences were noted in the wear experienced by opposing enamel surfaces, with strength-gradient zirconia (incorporating 5Y-PSZ) causing greater enamel wear than second-generation 3Y-TZP-based zirconia. These findings suggest that the increased cubic phase content and reduced transformation toughening in high-translucency zirconias may affect their interaction with natural enamel antagonists, despite similar restoration wear profiles.

### Clinical significance

When selecting zirconia material in restorative dentistry, clinicians should consider not only esthetics and mechanical strength but also the potential for enamel wear. While high-translucency, strength-gradient zirconias offer improved optical properties, their increased potential for enamel wear may raise concerns, particularly in patients with parafunctional habits or limited enamel thickness. Clinicians are advised to evaluate occlusion carefully, ensure optimal surface finishing (e.g., polishing rather than glazing), and consider the material’s phase composition to achieve long-term clinical success and preserve natural dentition.

### Clinical implications

These findings emphasize the importance of individualized material selection in restorative treatment planning. In esthetically demanding cases, strength-gradient zirconias may provide visual advantages; however, their potential to increase enamel wear must be considered, especially in patients with bruxism, misaligned occlusion, or reduced enamel volume. Routine follow-up and occlusal assessments are recommended to monitor enamel wear over time. Implementing careful polishing protocols and possibly limiting the use of high-translucency zirconias in high-stress occlusal zones could help mitigate enamel wear risks and improve long-term outcomes.

## Data Availability

The data that support the findings of this study are available on request from the corresponding author.

## Abbreviations

3D: Three-dimensional
3Y-TZP: 3 mol% yttria-stabilized tetragonal zirconia polycrystal
4Y-PSZ: 4 mol% yttria-partially stabilized zirconia
5Y-PSZ: 5 mol% yttria-partially stabilized zirconia
CAD/CAM: Computer-aided design/Computer-aided manufacturing
CONSORT: Consolidated Standards of Reporting Trials
EXOCAD: Dental CAD/CAM software
FPD: Fixed partial denture
Micro-CT: Micro-computed tomography
RCT: Randomized controlled trial
RMS: Root mean square
STL: Stereolithography (file format)
TMJ: Temporomandibular joint
